# Multi-Hole Self-Expandable Metallic Stent for Malignant Distal Biliary Obstruction: A Literature Review

**DOI:** 10.3390/jcm15041410

**Published:** 2026-02-11

**Authors:** Koh Kitagawa, Shohei Asada, Jun-ichi Hanatani, Yuki Motokawa, Yui Osaki, Tomihiro Iwata, Akira Mitoro, Hitoshi Yoshiji

**Affiliations:** 1 Department of Gastroenterology, Nara Medical University, Nara 634-8521, Japan; 2 Division of Endoscopy, Nara Medical University, Nara 634-8521, Japan

**Keywords:** malignant biliary obstruction, multi-hole self-expandable metallic stent, covered metallic stent, uncovered metallic stent, partially covered metallic stent

## Abstract

Endoscopic biliary drainage using self-expanding metal stents (SEMSs) is a standard palliative therapy for cholangitis and obstructive jaundice caused by malignant distal biliary obstruction (MDBO). Fully-covered SEMSs (FC-SEMSs) prevent tumor ingrowth and provide longer patency; however, recent advances in chemotherapy have increased stent migration due to tumor shrinkage, resulting in reduced functional patency compared with uncovered SEMSs. Partially covered SEMSs can reduce migration but are often difficult to remove after deployment. In addition, adverse events such as acute pancreatitis and cholecystitis remain a concern with FC-SEMSs. To address these limitations, Dr. Kobayashi introduced a novel porous SEMS with multiple side holes in the covering membrane (MH-SEMSs) in 2019. This design allows limited bile duct epithelial ingrowth through side holes, providing anchorage while maintaining removability. The side-hole structure may also reduce cholecystitis and pancreatitis by preserving flow through the pancreatic and cystic duct orifices. Over five years since their introduction, clinical evidence supporting MH-SEMSs has steadily increased. This review summarizes current data and explores future perspectives for MH-SEMS use in MDBO management.

## 1. Introduction

Endoscopic biliary drainage using self-expanding metallic stents (SEMSs) is widely employed as a palliative treatment for cholangitis and obstructive jaundice caused by malignant distal biliary obstruction (MDBO) [1,2]. Historically, uncovered SEMSs (U-SEMSs) were the only available option [3,4,5,6]; however, since the early 2000s, fully-covered SEMSs (FC-SEMSs)—in which the stent body is coated with a polymer membrane such as silicone—have become increasingly utilized [7,8,9]. FC-SEMSs can prevent stent occlusion due to tumor ingrowth, and several studies have reported longer stent patency compared with U-SEMSs [7,10].

However, recent advances in chemotherapy for pancreaticobiliary malignancies have increased the likelihood of tumor shrinkage, thereby increasing the risk of stent migration [11,12,13,14]. Consequently, FC-SEMSs have not consistently demonstrated superiority over U-SEMSs, largely because of their higher migration rates [9,15]. Partially covered SEMSs (PC-SEMSs), which have uncovered ends designed to reduce migration, have also been reported to be useful [16,17,18,19]. Nevertheless, PC-SEMSs are generally difficult to remove once deployed. In pancreatic cancer, two main regimens have been widely used: one based primarily on gemcitabine and the other on fluorouracil [11,12]. In recent years, the usefulness of regimens incorporating nanoliposomal irinotecan has also been reported, with the median survival period for patients with unresectable pancreatic cancer approaching one year [20,21]. Progress in chemotherapy for biliary tract cancer has been even more remarkable. In addition to conventional regimens combining gemcitabine, cisplatin, and S1 [22,23,24,25], the efficacy of regimens incorporating durvalumab or pembrolizumab has been reported. These combination therapies with immune checkpoint inhibitors have improved median survival in patients with biliary tract cancer, with an increasing proportion achieving long-term survival of 2–3 years [13,14]. Consequently, the survival period of patients with MDBO is beginning to exceed the average patency period of SEMSs. Therefore, these patients are increasingly likely to develop recurrent biliary obstruction (RBO) during their lifetime and require SEMS replacement. In recent years, SEMSs have been required to achieve the conflicting goals of preventing migration while also allowing removal and replacement. In addition, the risk of post-procedural adverse events, particularly acute pancreatitis and cholecystitis following FC-SEMS placement, remains a significant clinical concern [26,27,28,29,30,31].

To address these limitations, Dr. Kobayashi developed a porous multi-hole SEMS (MH-SEMS) with multiple side holes in the covering membrane, first reported in 2019 [32]. This novel stent is designed to reduce migration despite being covered, as partial embedding of the bile duct epithelium into the stent body occurs through the side holes. Furthermore, the presence of these side holes may decrease the risk of cholecystitis and pancreatitis by preventing obstruction of the cystic duct and pancreatic duct orifices. More than five years after its introduction, clinical reports evaluating endoscopic biliary drainage with MH-SEMSs in patients with MDBO have begun to accumulate [33,34,35,36,37,38]. However, clinical outcomes have varied considerably across studies. This review summarizes the current evidence and discusses the future prospects of MH-SEMSs in the management of MDBO.

## 2. Methods

Two authors (K.K. and S.A.) conducted a comprehensive literature search on MH-SEMS using PubMed, covering the period from the 2019 report by Kobayashi et al. [32] to December 2025. The search was limited to English-language studies involving human participants and used the keywords (“muti hole”) AND (“stent” OR “biliary”). Studies on hilar biliary drainage and publications available only as abstracts were excluded. Reference lists of relevant articles were carefully reviewed to identify additional studies. All endoscopic images presented in the figures were obtained from cases in which MH-SEMS was placed at our institution. Written informed consent was obtained from all patients for the publication of these images.

## 3. Multi-Hole Metallic Stents

**Figure 1:**
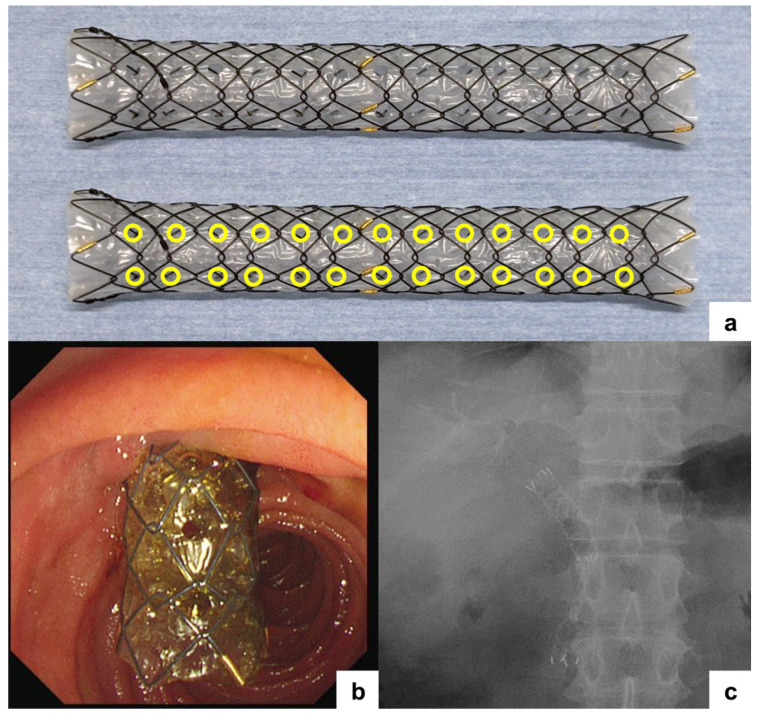
Structure and clinical placement of the multi-hole self-expandable metallic stent (MH-SEMS). (**a**) Image of the MH-SEMS showing six longitudinal rows of side holes in the covering membrane (yellow circles). Each side hole had a diameter of 1.8 mm. (**b**) Endoscopic view of MH-SEMS placement in a patient with a malignant distal biliary obstruction. (**c**) Corresponding fluoroscopic image confirming appropriate stent deployment.

The currently commercially available MH-SEMS is shown in Figure 1. The stent is manufactured by M.I. Tech (Pyeongtaek, Republic of Korea) and marketed as the HANAROSTENT^®^ Biliary Multi-hole^TM^ NEO. The stent body is constructed by braiding 0.0065-inch nitinol wire using a “hook & cross” technique, with a hook-to-cross ratio of 5:1. This structure provides a balanced combination of moderate radial expansion force and flexibility. The inner surface of the stent is covered with a silicone membrane, into which six longitudinal rows of side holes are incorporated (Figure 1, yellow circles). Each side hole has a diameter of 1.8 mm. MH-SEMSs are available in diameters of 8 mm and 10 mm, with lengths of 5, 6, 7, 8, and 10 cm. The stent is mounted on an 8-Fr delivery catheter with a total length of 180 cm, which is compatible with balloon-assisted endoscopic retrograde cholangiopancreatography (ERCP) in patients with surgically altered anatomy [39].

## 4. Stent Patency of Multi-Hole Metallic Stents

Stent migration is influenced by multiple factors, including the presence of flared ends, the radial expansion force of the stent body, and the physical properties of the wires used [40,41]. A conventional strategy to prevent migration of FC-SEMSs has been the use of PC-SEMSs, which have bare ends that allow embedding of the bile duct mucosa while maintaining central coverage to suppress tumor ingrowth. Although PC-SEMSs effectively prevent migration and maintain patency, they are essentially non-removable once deployed. With recent advances in chemotherapy for pancreatobiliary neoplasms leading to improved patient prognosis [11,12,13,14], the need for repeated endoscopic re-interventions has increased. Consequently, the use of non-removable PC-SEMSs has become less favorable in the palliative management of MDBO.

Although MH-SEMS is broadly categorized as PC-SEMS, it was developed based on a distinct concept and is designed to be removable when necessary. To date, seven studies evaluating MH-SEMSs in patients with MDBO have been identified through a PubMed search (Table 1) [32,33,34,35,36,37,38]. In 2019, Dr. Kobayashi first reported clinical outcomes using prototype MH-SEMSs in six patients [32]. These prototypes consisted of “small-hole” and “large-hole” designs, differing in side-hole size, and the small-hole type included a removal lasso. Among these initial cases, four involved malignant hilar biliary obstruction, one involved MDBO, and one involved a benign biliary condition.

Subsequently, Kulpatcharapong et al. reported outcomes of MH-SEMSs in a large cohort of patients with MDBO in Thailand [33]. This retrospective propensity score–matched study compared MH-SEMSs, FC-SEMSs, and U-SEMSs, demonstrating significantly longer patency in the MH-SEMSs, with a median time to recurrent biliary obstruction (TRBO) of 479 days. The authors attributed this favorable outcome to reduced migration compared with FC-SEMSs and reduced tumor ingrowth compared with U-SEMSs. In this report, the symptomatic stent migration rate for MH-SEMSs was 2.6%, which was significantly lower than the 15.8% rate observed for FC-SEMSs. However, it should be noted that the MH-SEMS used in this study differed from the currently marketed model. Early in the study period, first-generation MH-SEMSs with a greater number of side holes were used, but frequent tumor ingrowth through these holes prompted a transition to a second-generation design with approximately 30% fewer side holes.

From 2024 onward, reports focusing on the currently available MH-SEMS model began to emerge. Takeda et al. evaluated MH-SEMSs exclusively in patients with MDBO caused by unresectable pancreatic cancer, using a fully-covered HANARO stent as the comparator [34]. Contrary to earlier findings, MH-SEMSs demonstrated a significantly shorter patency period than FC-SEMSs, with a median TRBO of 101 days. The authors postulated that “small ingrowth” through the side holes may disturb antegrade bile flow, leading to sludge formation or non-obstructive cholangitis even without complete stent occlusion. They further suggested that the prolonged TRBO reported by Kulpatcharapong et al. may have been influenced by short follow-up duration and early censoring in the Kaplan–Meier analysis. Based on these observations, modification of the number and size of side holes was proposed. In contrast, this study also found that the stent migration rate with MH-SEMSs was significantly lower than that with FC-SEMSs (MH-SEMSs, 0% versus FC-SEMSs, 17.6%). Therefore, the antimigration performance of MH-SEMSs was consistent with the findings of Kulpatcharapong et al.

By 2025, additional studies further expanded the evidence base. Matsumoto et al. compared MH-SEMSs with FC-SEMSs in patients with MDBO and found a median TRBO of 151 days for MH-SEMSs, which was slightly shorter but not significantly different from that of FC-SEMSs [38]. In contrast, Takahashi et al. conducted a large multicenter retrospective study involving 111 patients, representing the largest MH-SEMS cohort to date [35]. The incidence of RBO during follow-up was 21%, with a median TRBO of 446 days, comparable to the results reported by Kulpatcharapong et al. Notably, symptomatic stent migration occurred in only two cases (1.9%).

Conversely, we recently reported two additional studies evaluating MH-SEMSs [36,37]. In the first, outcomes of MH-SEMSs were compared with conventional PC-SEMSs in patients with unresectable pancreatic cancer–related MDBO [36]. MH-SEMSs demonstrated an antimigration effect comparable to that of PC-SEMSs (migration rate: MH-SEMS, 4.8% versus PC-SEMSs, 3.2%); however, stent patency tended to be shorter, although the difference was not statistically significant. The most common cause of RBO was sludge formation, and the median TRBO was 318 days, intermediate between values reported by Takahashi et al. and Takeda et al. In our second report, MH-SEMSs were evaluated for preoperative biliary drainage in patients with resectable or borderline resectable pancreatic cancer undergoing neoadjuvant chemoradiotherapy [37]. Among 14 patients, the median time to surgery was 105 days, with only one case of RBO due to sludge. Despite tumor shrinkage, no stent migration was observed, and pathological assessment confirmed that MH-SEMS removal did not compromise diagnostic accuracy. Conversely, in our two studies evaluating MH-SEMSs in patients with MDBO caused by pancreatic cancer, sludge obstruction was the most frequent cause of RBO. This observation supports the hypothesis proposed by Takeda et al., suggesting that minor tumor ingrowth can disrupt bile flow and promote sludge accumulation.

Accordingly, reported patency periods of MH-SEMSs vary widely among studies. Nevertheless, there is consistent agreement that MH-SEMS is associated with a low incidence of stent migration. Among the seven studies on MH-SEMS, three directly compared it with FC-SEMS (Table 1) [33,34,38]. Regarding the incidence of adverse events, including pancreatitis and cholecystitis, none reported significant differences between the two stent types. However, findings regarding stent patency were inconsistent: one study reported no difference between MH-SEMSs and FC-SEMSs [38], one reported longer patency with MH-SEMSs [33], and one reported shorter patency with MH-SEMS [34]. Studies with larger sample sizes and heterogeneous malignancies tend to report longer TRBO, whereas studies limited to pancreatic cancer often demonstrate shorter TRBO. Pancreatic cancer–associated MDBO often involves duodenal invasion, which may impair duodenal peristalsis and predispose patients to sludge formation or non-obstructive cholangitis caused by “small ingrowth.” Consistent with this mechanism, our comparative study of MH-SEMSs and PC-SEMSs identified duodenal invasion in pancreatic cancer as an independent risk factor for shorter TRBO on multivariate analysis [36].

## 5. Removability of Multi-Hole Metallic Stents

**Figure 2:**
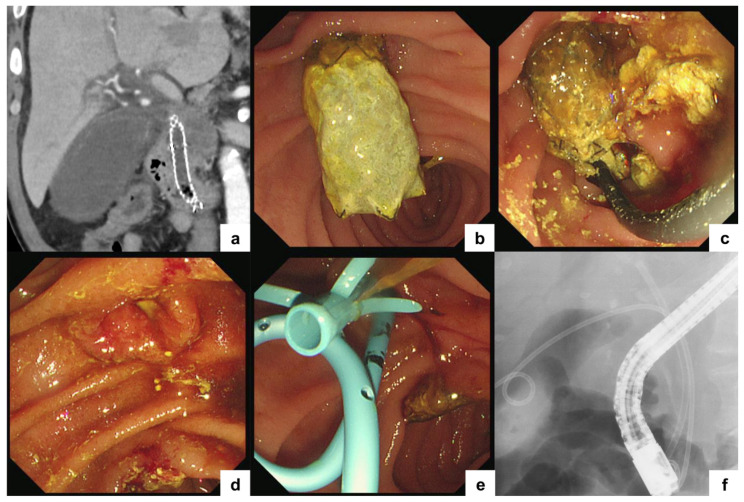
Endoscopic removal of a multi-hole self-expandable metallic stent (MH-SEMS). A patient with malignant distal biliary obstruction due to pancreatic cancer underwent MH-SEMS placement. (**a**) Computed tomography image obtained one month after stent placement demonstrating acute cholecystitis. Because of a bleeding tendency, percutaneous transhepatic biliary drainage and endoscopic ultrasound-guided gallbladder drainage were contraindicated. (**b**) Endoscopic view of the indwelling MH-SEMS. (**c**,**d**) Endoscopic images demonstrating MH-SEMS removal. The stent was grasped with a snare and successfully retrieved without any adverse events. (**e**,**f**) Plastic stents were subsequently placed in the bile duct and gallbladder.

Removal of SEMSs is commonly performed in cases of FC-SEMS occlusion or in the management of severe adverse events such as cholecystitis or pancreatitis [42]. However, evidence regarding the safety and feasibility of MH-SEMS removal remains limited. As summarized in Table 1, published reports suggest that MH-SEMS removal is generally feasible and safe. Nonetheless, publication bias should be considered, as unsuccessful or complicated removal cases may be underreported. While bile duct mucosal embedding into the side holes of the MH-SEMSs is minimal, stent removal is typically straightforward, as previously described (Figure 2). In contrast, prolonged indwelling time, damage to the cover membrane, or extensive tumor ingrowth involving the metallic framework may significantly complicate removal. Even in such scenarios, placement of an FC-SEMS within the MH-SEMS may facilitate removal using the stent-in-stent technique [43,44,45].

## 6. Re-Intervention for Recurrent Biliary Obstruction of Multi-Hole Metallic Stents

**Figure 3:**
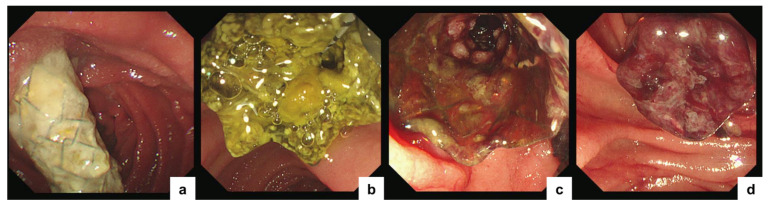
Endoscopic findings of recurrent biliary obstruction following multi-hole self-expandable metallic stent placement. (**a**) Partial stent migration. (**b**) Stent occlusion due to sludge accumulation. (**c**) Tumor ingrowth through the side holes. (**d**) Hemobilia originating from the side holes.

Re-intervention following FC-SEMS occlusion generally involves one of two strategies: (1) removal of the occluded stent followed by placement of a new stent, or (2) deployment of an additional stent within the existing stent [42]. The optimal approach depends primarily on the underlying cause of RBO (Figure 3). However, there is no consensus regarding the optimal stent type for re-intervention. If the initially placed MH-SEMSs demonstrated adequate long-term patency, reinsertion of the same device may be reasonable. Conversely, in cases of early occlusion or tumor-related hemorrhage, switching to a conventional FC-SEMS may be preferable. At our facility, MH-SEMS removal and replacement is performed when dislocation occurs or when sludge obstruction predominates with minimal tumor ingrowth. In contrast, in cases with significant tumor ingrowth or hemorrhagic complications, forced removal is avoided, and an FC-SEMS is deployed within the MH-SEMS using the stent-in-stent technique [43,44,45]. In this setting, the anchoring effect of the outer MH-SEMSs may reduce migration of the inner FC-SEMSs. When transpapillary re-intervention is not feasible, alternative approaches such as endoscopic ultrasound-guided biliary drainage (EUS-BD) should be considered. Indeed, the development of stents specifically for EUS-BD is progressing, as has been observed for stents used in MDBO, and EUS-BD may become the first-line option for biliary drainage in patients with MDBO [46,47,48,49].

## 7. Preventive Effect of Multi-Hole Metallic Stents on Post-Placement Pancreatitis and Cholecystitis

The unique side-hole design of MH-SEMSs was hypothesized to confer benefits beyond migration prevention, specifically, by preserving flow through the pancreatic duct and cystic duct and thereby reducing post-procedural pancreatitis and cholecystitis. However, published data do not consistently support this expectation. Across prior reports, the incidence of post-placement acute pancreatitis with MH-SEMSs is approximately 5–10% (Table 1), comparable to rates reported for other FC-SEMSs [31,50,51,52,53,54,55,56]. Notably, four comparative studies found no significant difference in pancreatitis incidence between MH-SEMS and competing stents [33,34,36,38].

In contrast, our study in patients with resectable pancreatic cancer (R-PC) showed a higher incidence of pancreatitis at 21.4% [37]. Because resectable tumors are typically smaller, many patients in this cohort likely had preserved pancreatic duct patency and pancreatic parenchymal volume, increasing their susceptibility to pancreatitis. Furthermore, in this MH-SEMS study, which exclusively targeted R-PC, endoscopic sphincterotomy (EST) was not performed in most cases, and rectal administration of indomethacin was also not performed. Based on these findings, we suggest that modifications such as reducing stent diameter may be necessary when MH-SEMS is used for preoperative biliary drainage in pancreatic cancer. Consistent with this need, a 6 mm-diameter MH-SEMS has recently been developed.

Cholecystitis following FC-SEMS placement is generally reported in 5–20% of cases [26,27,28,29,30,31]. Established risk factors include tumor invasion of the cystic duct, gallstones, and common bile duct diameter [27,28,31]. Reports on MH-SEMS indicate a cholecystitis incidence of approximately 5% (Table 1), suggesting a trend toward lower rates compared with conventional FC-SEMSs. However, in comparative studies, no significant difference in cholecystitis incidence was observed between MH-SEMSs and other stent types [33,34,36,38]. Notably, our 2025 study specifically examined risk factors for cholecystitis following MH-SEMS placement and found that MH-SEMS use itself did not confer protection [36]. Tumor invasion of the cystic duct was identified as the sole independent risk factor. Importantly, among patients with MDBO without cystic duct invasion of tumor, no cases of cholecystitis occurred after MH-SEMS placement.

Overall, current evidence does not clearly demonstrate a preventive effect of MH-SEMS on post-placement pancreatitis. The incidence of post-ERCP pancreatitis is not solely determined by the type of SEMS. It is also influenced by patient factors such as age, gender, and primary cancer type as well as procedural factors including pancreatic duct stent placement, EST, and the use of pancreatic duct guidewires [57,58,59,60,61]. Therefore, the side holes of MH-SEMS are unlikely to play a role in preventing post-stent placement pancreatitis. Furthermore, while MH-SEMSs may reduce cholecystitis risk in selected patients, this effect appears limited. Larger studies with detailed subgroup analyses are required to clarify these potential benefits.

## 8. Discussion

MH-SEMS may be particularly advantageous in patients with malignant pancreaticobiliary tumors undergoing aggressive chemotherapy, for whom stent migration is a major concern. However, MH-SEMS use may be contraindicated in cases where bleeding from the side holes is a risk, such as in hypervascular tumors. For patients receiving best supportive care without active chemotherapy, conventional FC-SEMS may remain preferable. Currently, there is no evidence that MH-SEMS prevents post-stent placement pancreatitis or cholecystitis. Further research is needed to test the hypothesis that the side holes of MH-SEMS help preserve flow through the pancreatic and cystic duct orifices.

Current evidence regarding MH-SEMSs is derived predominantly from retrospective studies. The type of tumor, presence of duodenal invasion, use of chemotherapy, stent generation and design, follow-up period, and even the definitions of outcomes (TRBO/RBO and their causes) vary across studies. Bias from these confounding factors may contribute to the variability in MH-SEMS patency observed above. To establish its true clinical value, large-scale, multicenter prospective studies are warranted. In contrast, further refinement of the MH-SEMS design may also be necessary, including optimization of side-hole size, number, and configuration. Such modifications may influence stent expandability and flexibility, highlighting the importance of complementary bench-top mechanical evaluations. Finally, the clinical utility of newly developed thinner-diameter MH-SEMS should be validated in future studies involving patients with MDBO.

## 9. Conclusions

MH-SEMS is a PC-SEMS with a low risk of migration and potential removability, making it a novel option for endoscopic biliary drainage in patients with MDBO.

## Figures and Tables

**Table 1:** Summary of studies on the clinical outcomes of MH-SEMS for malignant distal biliary obstruction (MDBO).

Author (year)	No. of MH-SEMS Cases	Study Design	Competing SEMS	Center	Median Follow-Up Periods	Primary Disease, *n*	Rate of Anti-cancer Treatment Received Cases	Median TRBO	Incidence Rate of PEP	Incidence Rate of Cholecystitis	Incidence Rate of RBO	Incidence Rate of Stent Migration	Most Common Reason of RBO	Success Rate of MH-SEMS Removal, *(n/N)*	Results of the Comparative Study on TRBO
Kobayashi [32] (2019)	1	Case report	N/D	Single	N/D	UR-PC 1	N/D	223 days	0%	0%	100%	0%	N/D	100% (1/1)	N/D
Kulpatcharapong et al. [33] (2024)	40	Retrospective comparative study	FC-SEMS, U-SEMS	Single	177 days	UR-PC 23; Other malignancies 17	42.5%	479 days	7.5%	5.3%	21.1%	2.6% †	Tumor ingrowth	2 cases *	MH-SEMS > FC-SEMS, U-SEMS
Takeda et al. [34] (2024)	27	Retrospective comparative study	FC-SEMS	Single	N/D	UR-PC only	85.2%	101 days	3.7%	0%	53.8%	0% †	Non-occlusion cholangitis	100% (11/11)	MH-SEMS < FC-SEMS
Matsumoto et al. [38] (2025)	26	Retrospective comparative study	FC-SEMS	Single	103 days	UR-PC 22; Other malignancies 4	N/D	151 days	3.9%	0%	7.7%	3.8%	Food impaction, migration	N/D	MH-SEMS = FC-SEMS
Takahashi et al. [35] (2025)	111	Retrospective study	N/D	Multicenter	107 days	R/UR-PC 85; Other malignancies 26	60.4%	446 days	11.7%	2.7%	21.0%	1.9%	Tumor ingrowth, Non-occlusion cholangitis	88.9% (16/18)	N/D
Asada et al. [36] (2025)	43	Retrospective comparative study	PC-SEMS	Single	177 days	UR-PC only	62.8%	318 days	4.8%	7.1%	28.6%	4.8% ‡	Sludge occlusion	100% (7/7)	MH-SEMS = PC-SEMS
Asada et al. [37] (2025)	14	Retrospective study	N/D	Single	N/D	R/BR-PC only	100% (NACRT)	N/D	21.4%	0%	7.1%	0%	Sludge occlusion	100% (1/1)	N/D

SEMS, self-expandable metallic stent; MH-SEMS, multi-hole self-expandable metallic stent; FC-SEMS, fully-covered self-expandable metallic stent; U-SEMS, uncovered self-expandable metallic stent; PC-SEMS, partially covered self-expandable metallic stent; TRBO, time to recurrent biliary obstruction; PEP, post-endoscopic retrograde cholangiopancreatography pancreatitis; UR-PC, unresectable pancreatic cancer; R-PC, resectable pancreatic cancer; BR-PC, borderline resectable pancreatic cancer; NACRT, neoadjuvant chemoradiation therapy; N/D, not described; * The success rate of MH-SEMS removal is not described; † The migration rate of MH-SEMS was significantly lower than that of FC-SEMS. ‡ The migration rate of MH-SEMS was equivalent to that of PC-SEMS.

## Data Availability

No new data were created or analyzed in this study.

## Abbreviations

The following abbreviations are used in this manuscript:

MDBO	Malignant distal biliary obstruction
MH	Multi-hole
SEMS	Self-expanding metal stent
TRBO	Time to recurrent biliary obstruction
